# *Cnidium officinale* extract and butylidenephthalide inhibits retinal neovascularization in vitro and in vivo

**DOI:** 10.1186/s12906-016-1216-8

**Published:** 2016-07-19

**Authors:** Yun Mi Lee, Yu-Ri Lee, Chan-Sik Kim, Kyuhyung Jo, Eunjin Sohn, Jin Sook Kim, Junghyun Kim

**Affiliations:** Korean Medicine Convergence Research Division, Korea Institute of Oriental Medicine (KIOM), 1672 Yuseongdaero, Yuseong-gu, Daejeon, 34054 South Korea; Department of Biology, Chungnam National University, Daejeon, 34134 South Korea

**Keywords:** Retinal neovascularization, Butylidenephthalide, *Cnidium officinale*, Oxygen-induced retinopathy, Vascular endothelial growth factor

## Abstract

**Background:**

Retinal neovascularization, which is the pathological growth of new blood vessels, is associated with retinopathy of prematurity, neovascular age-related macular degeneration, diabetic retinopathy and retinal vein occlusion. In this study, we evaluated the effect of an extract of *Cnidium officinale* Makino (COE) and its bioactive compound, butylidenephthalide (BP), on the migration and tube formation of human umbilical vein endothelial cells (HUVECs), and on retinal pathogenic neovascularization in the oxygen-induced retinopathy (OIR) mouse model*.*

**Method:**

The HUVECs were incubated with COE and BP (0.1–10 μg/ml). The mice were exposed to 75 % oxygen for 5 days starting on the 7^th^ postnatal day (P7–P12). Then, the mice were returned to room air and intraperitoneally injected with COE (100 mg/kg) and BP (5 mg/kg) once per day for 5 days (P12–P16). On P17, we measured retinal neovascularization and analyzed the angiogenesis-related proteins expression using protein arrays.

**Results:**

COE and BP inhibit the HUVECs migration and the tube formation in a dose-dependent manner. In addition, COE significantly decreased retinal neovascularization in the OIR mice. COE reduced the expression levels of AREG, ANG, DLL4, Endostatin, IGFBP-2 and VEGF. Additionally, BP also inhibited the retinal neovascularization and down-regulated the expression of AREG, ANG, DLL4 and VEGF.

**Conclusion:**

These results suggest that COE and BP exerts antiangiogenic effects on retinal neovascularization by inhibiting the expression of AREG, ANG, DLL4 and VEGF, indicating that antiangiogenic activities of COE may be in part due to its bioactive compound, BP.

## Background

Retinal pathological neovascularization is a central feature of retinopathy of prematurity, neovascular age-related macular degeneration, diabetic retinopathy and retinal vein occlusion and is the leading cause of irreversible blindness globally [1].

Vascular endothelial growth factor (VEGF) is a major proangiogenic factor in retinal neovascularization caused by hypoxia-induced retinal damage. Hypoxia regulates sprouting angiogenesis and various proangiogenic growth factors through multiple pathways and increases VEGF expression at the level of gene transcription, mRNA stability, translation, and protein secretion levels [2, 3].

*Cnidium officinale* Makino, which belongs to the Umbelliferae family, has been used as a traditional oriental medicine in East Asian countries. The dried rhizomes of *C. officinale* have been used in the treatment of pain, anti-vitamin deficiency disease, menstrual disturbance, inflammation and as a blood pressure depressant. In addition, there are several reports suggesting that the dried rhizomes of *C. officinale* have multifunctional properties in tumor metastasis [4], angiogenesis [5], inflammation [6] and that they have antioxidant effects [7]. Butylidenephthalide (BP) is an alkylphthalide derived from the volatile oil of *C. officinale.* BP has antiangiogenic activities, which are associated with the activation of the p38 and ERK1/2 signaling pathways [8]. BP has been identified as having a variety of potential pharmacological activities, such as anti-cancer [9], anti-inflammatory [10], anti-platelet [11], vasorelaxant [12], anti-anginal [13] and anti-atherosclerotic [14] effects.

We have previously reported that Samul-tang; a mixture of four herbs, including *Angelicae gigantis*, *Cnidium officinale*, *Paeonia lactiflora*, and *Rehmannia glutinosa*; has an inhibitory effect on the retinal pathogenic angiogenesis induced by ischemic retinopathy in OIR mice [15]. Although the antiangiogenic properties of *C. officinale* and its bioactive ingredient, BP, have been reported, the effect on retinal pathogenic neovascularization in the oxygen-induced retinopathy (OIR) mouse model is still unknown. Therefore, in this study, we investigated the inhibitory effect of the extract of *C. officinale*, one of the four components of Samul-tang and its bioactive compound, BP on the migration and tube formation by HUVECs, and on retinal pathogenic neovascularization in the oxygen-induced retinopathy (OIR) mouse model.

## Methods

### Preparation of *Cnidium officinale*

A standardized COE was purchased from a plant extract bank at the Korea Research Institute of Bioscience & Biotechnology (Daejeon, Korea). A collection of voucher specimens is available for confirmation in the plant extract bank (Korea Research Institute of Bioscience and Biotechnology, Daejeon, Korea). Briefly, dried and grinded *C. officinale* rhizomes (4.6875 g) waere boiled with distilled water at 100 °C for 2 h, and the extract was filtered, lyophilized (yield: 33.3 %). COE was standardized using a reference compounds, butylidenephthalide (Sigma, MO, USA), by high performance liquid chromatography (HPLC).

### The wound healing migration assay

Human umbilical vein endothelial cells (HUVECs) were purchased from Cell Systems (Kirkland, WA, USA) and were taken from passages 3 to 6 in this study. HUVECs were seeded at 3.5 × 10^5^ cells/well in 12-well culture plates (Nalgene Nunc, NY, USA) and cultured in medium for 24 h. Confluent HUVECs were wounded with pipette tips. After the wounding, the plates were rinsed with a serum-free medium, and treated with COE (0.1–10 μg/ml) or BP (0.1–10 μg/ml) for 12 h. The cells were observed under a light microscope (BX51; Olympus, Tokyo, Japan) and photographed in triplicate at 200× magnification. Cell migration was determined by counting the number of cells that moved beyond the reference line in randomly selected fields.

### The tube formation assay

Tissue culture plates were coated with 400 μL of growth factor–reduced basement membrane matrix (Matrigel; Becton Dickenson, Franklin Lakes, NJ, USA). HUVECs were seeded at 1 × 10^5^ cells/well in 24-well culture plates (Nalgene Nunc, NY, USA) and treated with EGM-2 media containing COE (0.1–10 μg/ml) or BP (0.1–10 μg/ml) for 17 h. The tubes formed was observed under a light microscope and photographed at 200× magnification. Tube formation was quantified by manually counting the branching points of capillary-like structures per visual field.

### An oxygen-induced retinopathy(OIR) mouse model

Ischemic retinopathy was induced in C57BL/6 mouse pups, as previously described [16]. On the 12^th^ postnatal day (P12), after being exposed to 75 ± 2 % oxygen for 5 days (P7–P12), the mice were randomly assigned to one of three groups: the OIR, COE (100 mg/kg/day) or BP (5 mg/kg/day) group. The normal control group (Nor) was maintained under room air from birth until the 17^th^ postnatal day (P0 to P17). To minimize the differences in the weights of the pups, one mouse nursed 6–8 pups, and low-weight pups were discarded from the data sets. The COE and BP were dissolved in 5 % DMSO-containing saline immediately before use, and 100 μl of this solution was injected intraperitoneally once per day for 5 days (P12–P16). The Nor and OIR groups were injected with same volume of vehicle solution for 5 days. On P17, after 5 days of intraperitoneal injections, the mice were anesthetized and sacrificed. These experiments were repeated four times using four animals in each group. In our previous study, COE and BP prevented subretinal neovascularization in an animal model of laser-induced choroidal neovascularization [17]. The effective doses of COE and BP were 100 mg/kg and 5 mg/kg, repectively. Based on our previous findings, the doses of COE and BP in OIR mice were determined. All experiments that used animals were approved by the Institutional Animal Care and Use Committee of the Korea Institute of Oriental Medicine (Daejeon, Korea).

### Fluorescein-dextran microscopy and lectin staining

On P17, the mice in each group were anesthetized with zolazepam (Zoletil, Virbac, Carros, France). PBS containing fluorescein-dextran (FD40S, Sigma, MO, USA).was subsequently circulated through the left ventricle. The retinas were dissected, flat mounted onto glass slides and viewed using fluorescence microscopy (BX51, Olympus, Tokyo, Japan). The non-perfusion area in the retina were measured in flat mounts labeled with fluorescein-dextran The neovascular tufts in the retina were measured in flat mounts labeled with tetramethylrhodamine isothiocyanate (TRITC)-conjugated isolectin B4 (Sigma, MO, USA). The retinas were incubated with 1 % bovine serum albumin and 5 % Triton X-100 in PBS for 3 hours at room temperature.The retinas were washed 3 times with PBS and incubated overnight at 4 °C with *Bandeiraea simplicifolia* isolectin B4 (Sigma-Aldrich, MO, USA) diluted 1:50 in PBS. The retinas were washed with 0.05 % Tween 20 in PBS, followed by incubation with streptavidin TRITC (1:500, Serotec, Oxford, UK) for 4 h at 37 °C. The neovascular tufts was viewed with an Olympus BX51 microscope (Olympus, Tokyo, Japan). Quantification of the non-perfusion area and neovascular tufts in the retina was quantified using ImageJ software (NIH, MD, USA).

### Angiogenesis-related protein array

To investigate angiogenesis-related proteins in the retinas, a mouse angiogenesis array (R&D Systems, MN, USA) was performed according to the manufacturer’s protocol. Briefly, the retinas (*n* = 3) were homogenized in PBS using protease inhibitors and centrifuged at 10,000 x g for 5 minutes, and 250 ug of total protein concentrations were quantified. The lysates were added to a membrane spotted with antibodies against angiogenesis-related proteins. After being incubated overnight at 4 °C, the membranes were treated with streptavidin-horseradish peroxidase and were visualized using an enhanced chemiluminescence detection system (Amersham Bioscience, NJ, USA) on an image analyzer (LAS-3000, Fujifilm, Tokyo, Japan). Optical density measurements were obtained using the ImageJ software (NIH, MD, USA).

### Real-time PCR analysis

Total RNA was isolated using the TRIzol reagent (Invitrogen, CA, USA), and 0.5 μg of total RNA was reverse transcribed into cDNA with the PrimeScript First Strand cDNA Synthesis kit (Bio-Rad, CA, USA). Real-time quantitative PCR was performed with specific primers for VEGF and GAPDH using an iQ5 Continuous Fluorescence Detector System (Bio-Rad, CA, USA). The sequences of the primers were as follows: for VEGF, 5′-TCC TCC TAT CTC CAC CAC CTA TCC-3′ and 5′-GAC CCA GCC AGC CAT ACC C-3′; and for GAPDH, 5′-AAC GAC CCC TTC ATT GAC-3′ and 5′-TCC ACG ACA TAC TCA GCA C-3′. All real-time PCR experiments were run in triplicate. The mRNA levels of GAPDH were determined for the normalization of the VEGF mRNA expression values using the iQ5 optical system software (Bio-Rad, CA, USA).

### Statistical analysis

The results were expressed as the mean ± SE and were analyzed using one-way analysis of variance (ANOVA) followed by Tukey’s multiple comparison tests or using unpaired Student’s *t-*tests. All analyses were performed using the *Prism 6.0* software (GraphPad Software, San Diego, CA, USA).

## Results

### COE and BP inhibits migration and tube formation of HUVECs

**Fig. 1:**
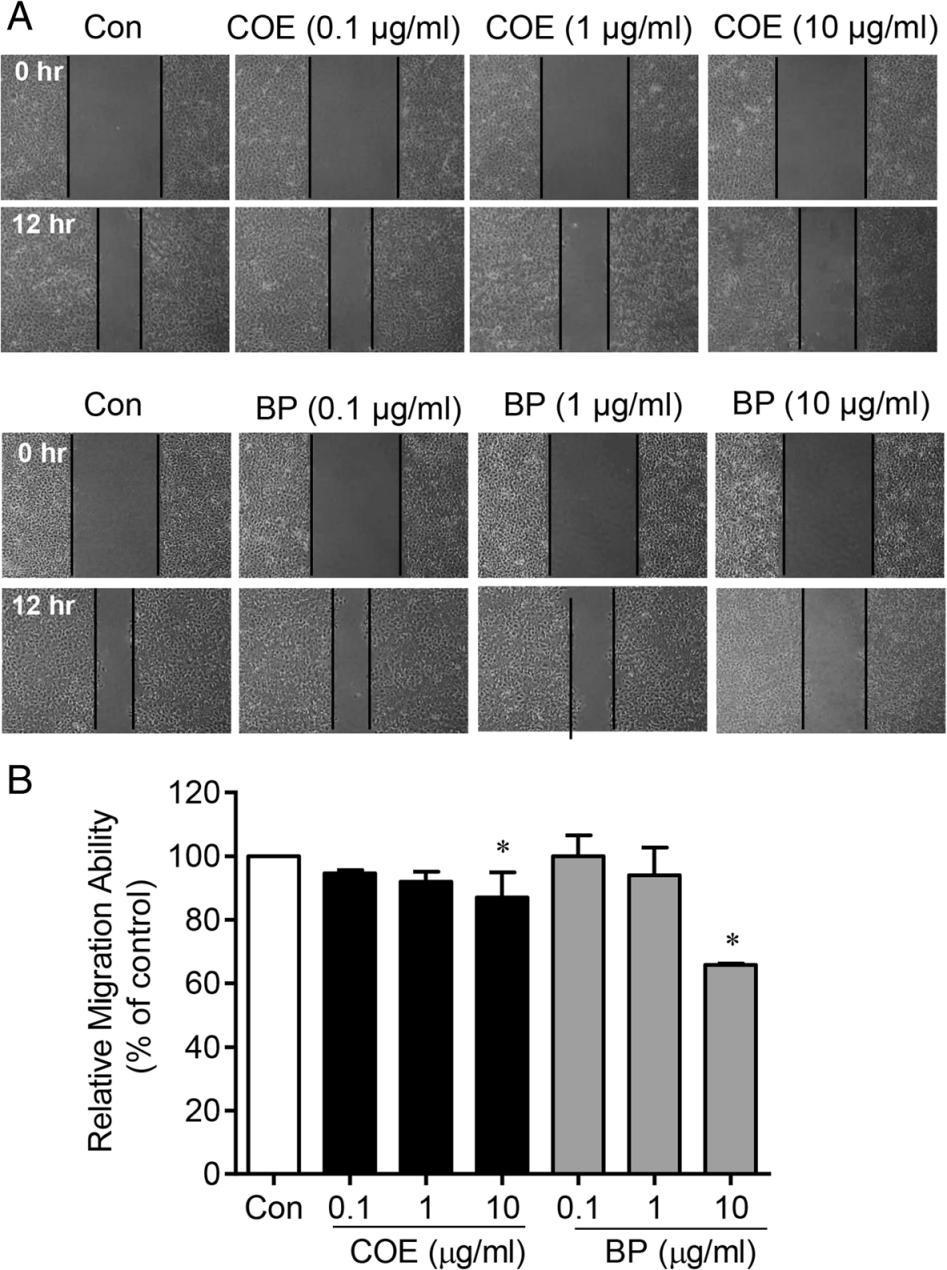
The effect of COE and BP on the migration of human umbilical vein endothelial cells. **a** COE and BP suppressed migration in HUVECs in the wound healing assay. The cells were treated with COE and BP and incubated at 37 °C. After 12 h, the number of cells that had migrated into the scratched area was calculated. **b** The bar graph represents the quantification of migrated cells from the untreated control, the COE-treated and the BP-treated groups. The data are presented as the mean ± SE. (*n* = 3; * *p* < 0.001 vs. the control group)

**Fig. 2:**
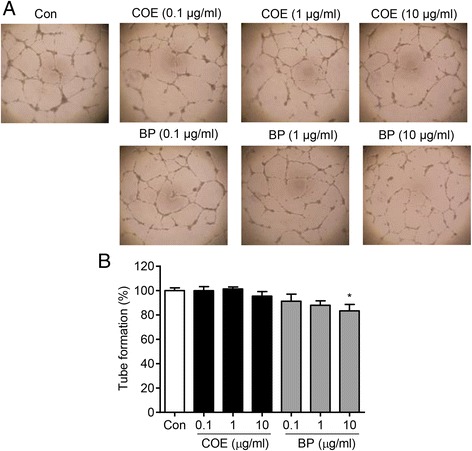
The effect of COE and BP on tube formation of human umbilical vein endothelial cells. **a** Matrigel (400 *μ*L) was added to 24-well plates and allowed to solidify for 1 h at 37 °C. HUVECs were treated with COE and BP in VEGF-containing EGM-2 media and incubated at 37 °C. After 17 h, randomly chosen fields were photographed under light microscope. **b** The bar graph represents the quantification of tube formation of the control group, the COE-treated and BP-treated groups. The data are presented as the mean ± SE. (*n* = 3; * *p* < 0.05 vs. the control group)

COE and BP have an inhibitory effect against the migration of HUVECs. As shown in Fig. 1, COE inhibited the HUVEC migration distance by approximately 94.6 ± 2.4 %, 92.0 ± 6.3 % and 87.0 ± 15.9 % at 0.1, 1 and 10 μg/ml doses, respectively. In addition, BP (10 μg/ml) also inhibited the HUVEC migration distance more potently than COE (65.76 ± 0.79 %). We next examined the inhibitory effects of COE and BP on the tube formation of HUVECs. The HUVECs plated on matrigel formed a massive network of tubes after 17 h, which was disrupted by COE and BP treatment (0.1–10 μg/ml). As shown in Fig. 2, COE treatment slightly changed the tube formation compared with the control group. However, BP treatment markly inhibited the tube formation by approximately 91.3 ± 16.2 %, 87.9 ± 9.8 % and 83.4 ± 14.7 % at 0.1, 1 and 10 μg/ml doses, respectively. In our unpublished data, the quantity of BP in COE were 0.21 ± 0.01 mg/g. Thus, the total BP content is 2.1 ± 0.1 ng/ml in 10 μg/ml of COE. This concentration for 10 μg/ml of BP is equal to 47 mg/ml of COE. Therefore, activities of COE at the concentration of 10 μg/ml were weaker than those of BP at same concentration in the endothelial cell migration assay and tube formation assay. These results clearly showed that COE and BP treatment inhibit motility and tubular structure formation as well as disrupts the preformed capillary tubes by HUVECs.

### COE inhibits retinal neovascularization

**Fig. 3:**
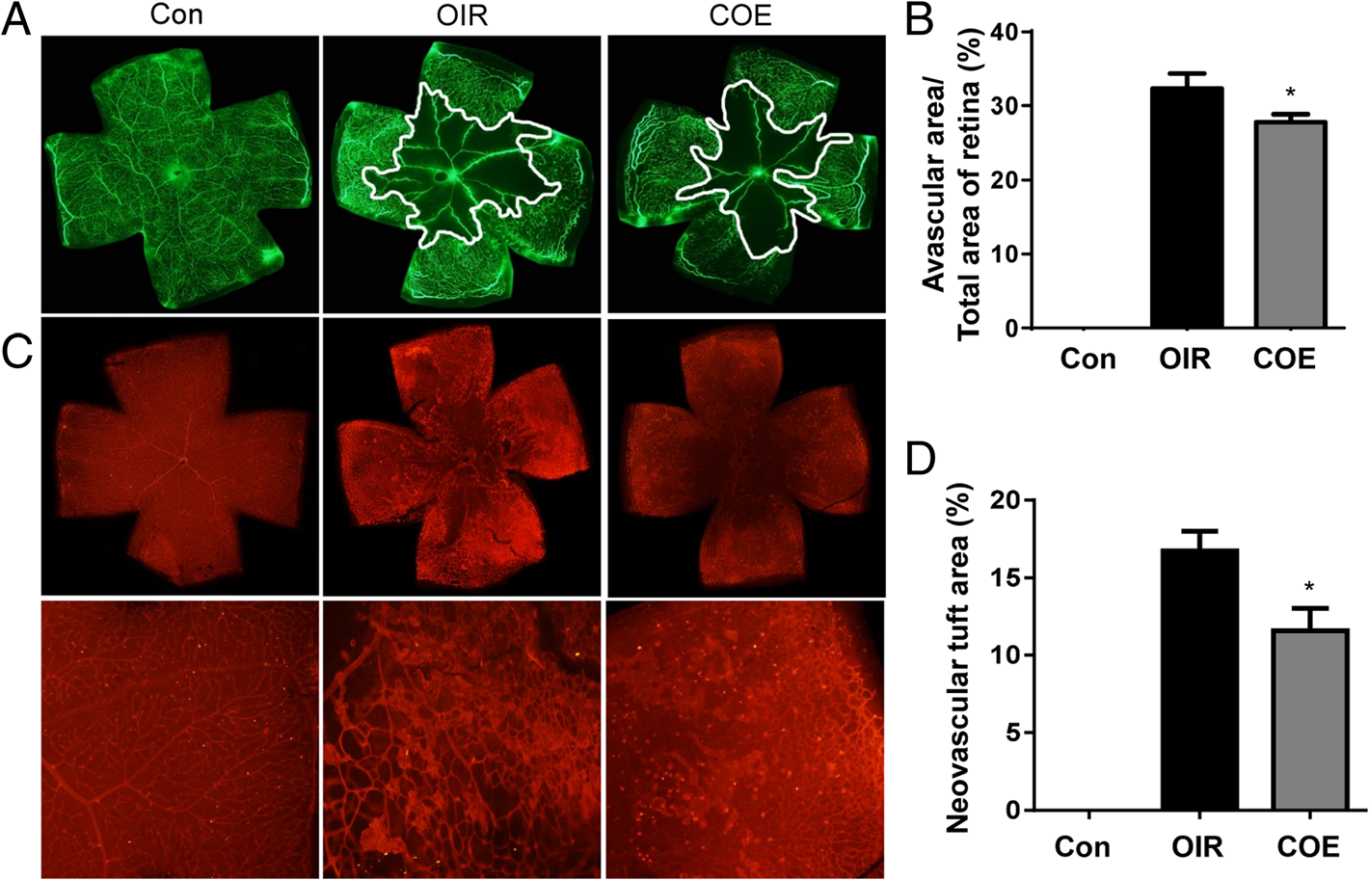
The effect of COE on retinal neovascularization in OIR mice. **a** The retinal blood vessels were visualized via fluorescein angiography using FITC-dextran. Nor, normal control mice; OIR, saline-treated OIR mice and COE, OIR mice treated with 100 mg/kg of COE. **b** The quantification results are expressed as the percentage of the central nonperfused area within the total retinal area. **c** The retinal neovascular tufts were visualized using isolectin B4 staining. Nor, normal control mice; OIR, saline-treated OIR mice; COE, OIR mice treated with 100 mg/kg of COE. **d** The quantification results are expressed as neovascular tufts on the retina surface. The bar graph values represent the mean ± SE (*n* = 5; *p < 0.05 vs. the saline-treated OIR mice)

The OIR mice that were treated with COE exhibited a significant decrease in the pathological changes that occurred during ischemic retinopathy. As shown in Fig. 3, COE decreased the area of vascular obliteration of the central retina and prevented pathogenic retinal neovascularization compared with the OIR group on P17. Mice that were treated with 100 mg/kg of COE had significantly decreased non-perfused areas compared with the OIR group (Fig. 3a). In addition, COE considerably decreased the formation of neovascular tufts (by 30.69 %) compared with the OIR group (Fig. 3c). Consequently, COE helped prevent the pathogenic retinal neovascularization during ischemic retinopathy.

### COE regulates the expression of angiogenesis-related factors

**Fig. 4:**
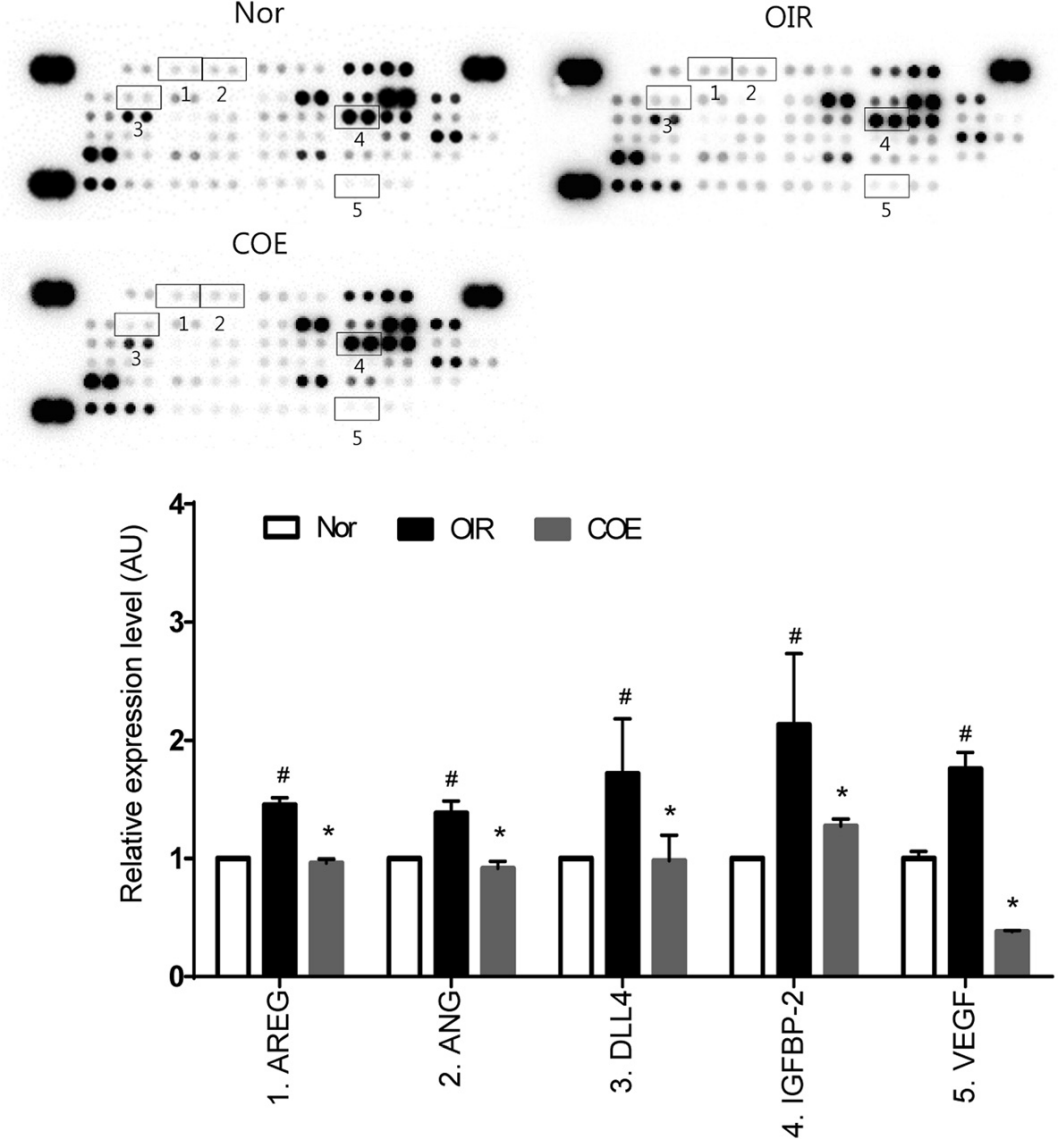
The expression of retinal angiogenesis-related proteins in OIR mice. Expression of proangiogenic and antiangiogenic factors in the retina were analyzed using protein arrays and quantified using ImageJ software. Proteins for which expression was modulated in COE-treated retinas are indicated by numbers. Nor, normal control mice; OIR, saline-treated OIR mice and COE, OIR mice treated with 100 mg/kg COE. 1: AREG, amphiregulin; 2: ANG, angiogenin; 3: DLL4, Delta like ligand 4; 4: IGFBP-2, insulin-like growth factor binding protein 2; 5: VEGF: Vascular endothelial growth factor. The bar graph values represent the mean ± SE (*n* = 3; ^#^ $p$ < 0.05 vs. the normal control mice, *p < 0.05 vs. the saline-treated OIR mice)

We investigated the expression levels of angiogenesis-related factors in the retina using a protein array to evaluate the direct effects of COE on OIR. As shown in Fig. 4, COE decreased the expression of proangiogenic factors (i.e., amphiregulin (AREG), angiogenin (ANG), delta like ligand 4 (DLL4), insulin-like growth factor binding protein-2 (IGFBP-2) and VEGF) in a dose-dependent manner compared with saline-treated OIR mice. These results indicate that COE might exert antiangiogenic effects by inhibiting the expression of AREG, ANG, DLL4, IGFBP-2 and VEGF.

### BP blocks retinal neovascularization

**Fig. 5:**
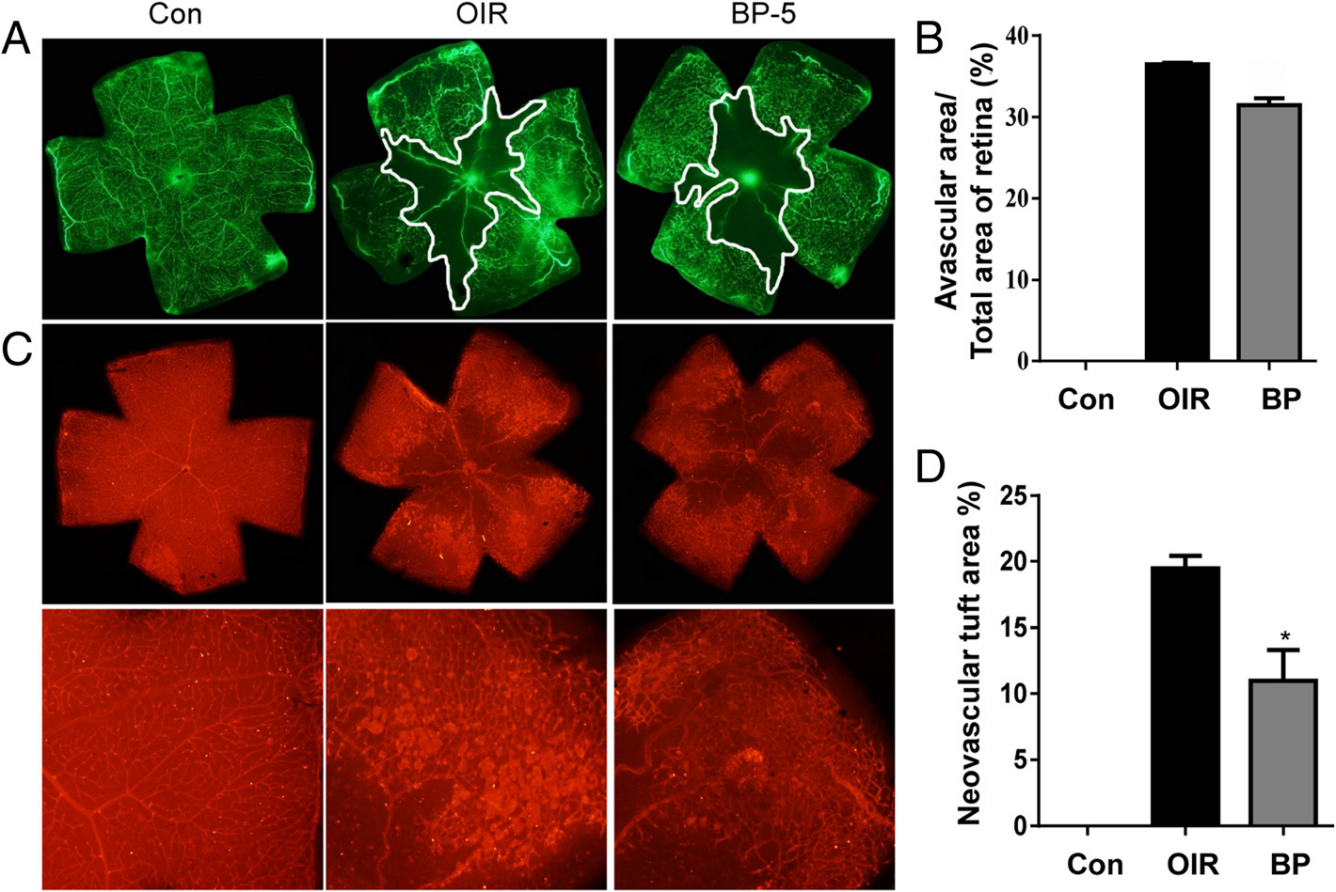
The effect of BP on retinal neovascularization in OIR mice. **a** The retinal blood vessels were visualized via fluorescein angiography using FITC-dextran. Nor, normal control mice; OIR, saline-treated OIR mice and BP, OIR mice treated with 5 mg/kg of BP. **b** The quantification results are expressed as the percentage of the central nonperfused area within the total retinal area. **c** The retinal neovascular tufts were visualized using isolectin B4 staining. Nor, normal control mice; OIR, saline-treated OIR mice and BP, OIR mice treated with 5 mg/kg of BP. **d** The quantification results are expressed as neovascular tufts on the retina surface. The bar graph values represent the mean ± SE (*n* = 5; *p < 0.01 vs. the saline-treated OIR mice)

BP is one of the major compounds found in *C. officinale*. To determine whether BP has a preventive effect against pathological retinal neovascularization, this compound was also administered in the OIR mouse model. As shown in Fig. 5, mice that were treated with 5 mg/kg of BP failed to exhibit a significant change in the area of non-perfusion in the center of the retina (Fig. 5a), but BP significantly reduced the formation of neovascular tufts (by 43.59 %) compared with the OIR group (Fig. 5c). These results indicate that BP is a potent antiangiogenic bioactive compound of *C. officinale*

### BP also regulates the expression of angiogenesis-related factors, similar to those observed in the COE-treated mice

**Fig. 6:**
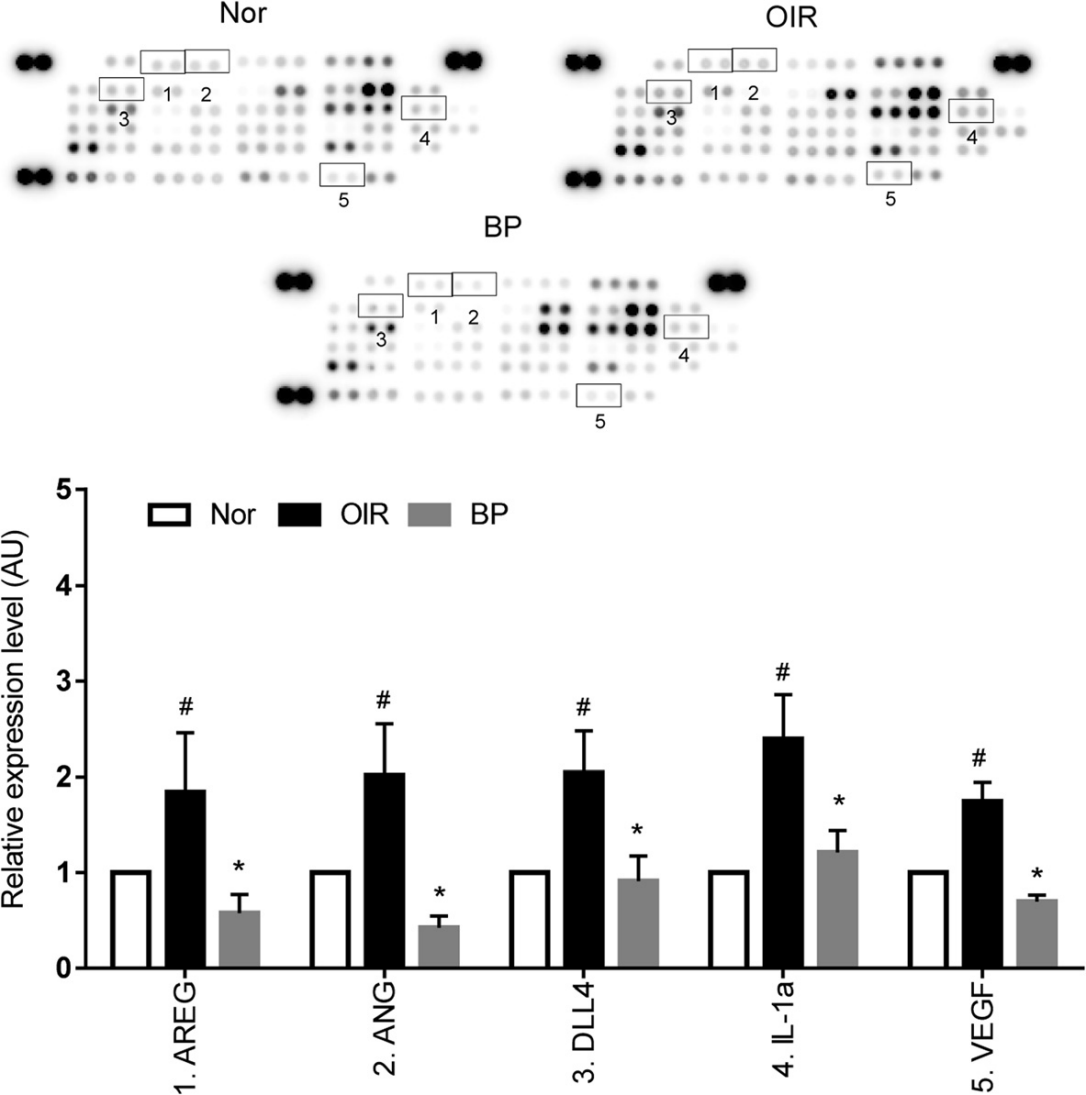
The expression of retinal angiogenesis-related proteins in OIR mice. Proteins for which expression was modulated in BP-treated retinas are indicated by numbers. Nor, normal control mice; OIR, saline-treated OIR mice and BP, OIR mice treated with 5 mg/kg of BP. 1: AREG, amphiregulin; 2: ANG, angiogenin; 3: DLL4, Delta like ligand 4; 4:IL-1α, interleukin-1 alpha; 5: VEGF: Vascular endothelial growth factor. The bar graph values represent the mean ± SE (*n* = 3; ^#^ $p$ < 0.05 vs. the normal control mice, *p < 0.05 vs. the saline-treated OIR mice)

In the protein array, BP decreased the expression of proangiogenic factors (i.e., AREG, ANG, DLL4, interleukin-1 alpha (IL-1α) and VEGF) in a dose-dependent manner compared with saline-treated OIR mice (Fig. 6). These results suggest that BP mediated antiangiogenic effects by inhibiting the expression of ARED, ANG, DLL4 and VEGF, indicating that the antiangiogenic activities of COE may be in part due to its bioactive compound, BP.

### COE and BP treatmemt downregulates VEGF mRNA expression

**Fig. 7:**
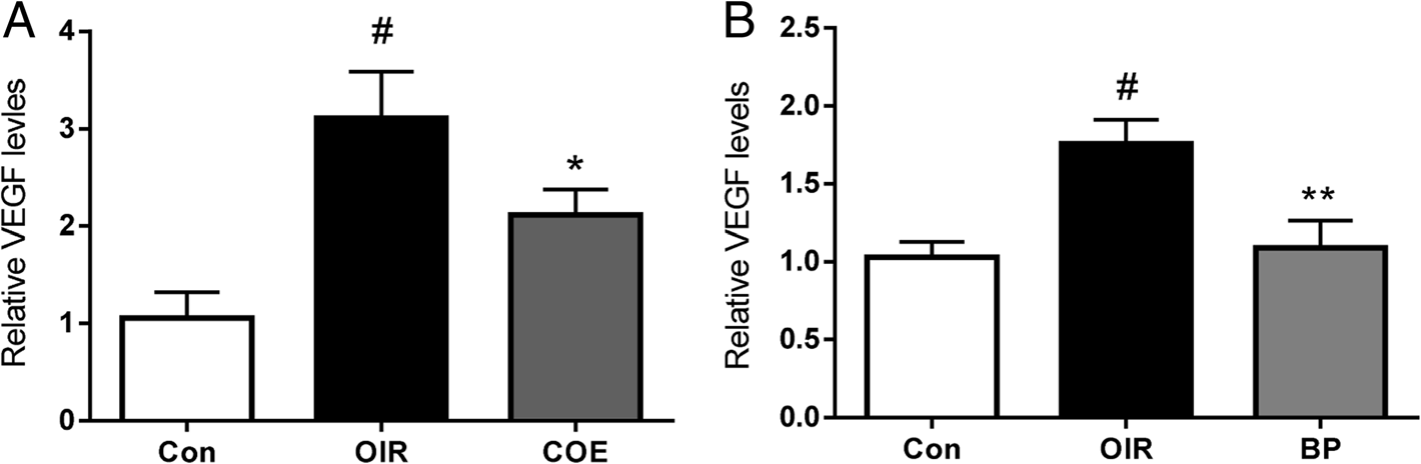
Real-time PCR analysis of VEGF mRNA levels in OIR mice. When compared with the normal controls, the VEGF group mRNAs were dramatically increased in the retinas of OIR mice and markedly reduced after COE (**a**) or BP (**b**) treatment. Nor, normal control mice; OIR, saline-treated OIR mice; COE, OIR mice treated with COE (100 mg/kg); and BP, OIR mice treated with BP (5 mg/kg). The data are shown as the mean ± SE (*n* = 6; ^#^ $p$ < 0.001 vs. the normal control mice, *p < 0.05, **p < 0.01 vs. the saline-treated OIR mice)

In antibody array, the expression levels of various angiogenic factors were changed by COE or butylidenephthalide. However, when considering 1.5-fold change thresholds, VEGF only displayed a >1.5-fold up-regulation in the vehicle-treated rats and a <1.5-fold down-regulation in the COE as well as butylidenephthalide-treated group. Thus, to confirm the change of VEGF expression levels in the retina, we examined the induction of VEGF mRNA using real-time PCR. As expected, we observed a robust induction of VEGF mRNA during oxygen-induced retinopathy. However, the VEGF levels were significantly decreased in COE and BP treated OIR mice (Fig. 7).

## Discussion

Retinal neovascularization is a major cause of blindness worldwide. Various therapeutic strategies such as laser photocoagulation, photodynamic therapy, and anti-VEGF therapy result in the regression of the retinal neovascularization. However, these retinal neovascularization therapies are limited and have severe undesirable side effects [18]. Thus, it is necessary to develop better retinal neovascularization treatment therapeutic targets. In the present study, we evaluated the antiangiogenic effects of *Cnidium officinale* and its bioactive compound, BP, during pathological retinal neovascularization in OIR mice.

The OIR model has been widely used as a valuable tool in retinal neovascularization pathogenesis research [19]. In the mouse OIR model, the hyperoxic phase (P7–P12) results in the loss of immature vessels in the central retina and the development of vaso-obliteration. When the mice are returned to room air (P12–P17), the retina becomes hypoxic due to the absence of the retinal vasculature, which stimulates proangiogenic factors and results in abnormal neovascularization [20]. Many previous studies using the OIR model have demonstrated the valuable effect of this model and have laid the foundation for today’s clinical application of antiangiogenic treatments in retinal neovascularization [19, 20].

The neovascular processes occurring in OIR include pathogenic preretinal neovascular formations and intraretinal revascularization. In our study, COE potently inhibited neovascular tuft formation and enhanced revascularization of deep retinal vascular plexuses. Although this accelerated retinal revascularization effect of COE that accompanied its potent angiostatic activity was surprising, such dual properties (angiostasis of pathologic neovascularization and facilitation of physiological revascularization) have been also shown in several anti-anigiogenic agents. Banin et al. showed that T2-TrpRS (carboxyl-terminal fragment of tryptophan tRNA Synthetase) that not only inhibit pathologic neovascularization, but also facilitate physiological revascularization of ischemic retinal tissue [21]. Wang et al. also showed that tenomodulin (a novel member of the tumor necrosis factor family) similarly reducing preretinal neovascular tuft formation and enhance revascularization of the obliterated areas [22]. The mechanism of dual actions of these agents remains unclear. Angiogenesis is a complex process regulated by an intricate balance of stimulators and inhibitors. Multiple signaling pathways converge to regulate the growth of new blood vessels during both physiological and pathologic neovascularization, and some factors have demonstrated seemingly conflicting, environmentally dependent roles in this complex process. Although both processes proceed by angiogenesis, physiological vascular development is highly controlled and occurs under moderate levels of hypoxia [23], whereas pathogenic neovascularization in OIR occurs in a highly hypoxic environment [24]. The apparent paradoxical effects of COE could be reflect different microenvironment-dependent activities. Another possible explanation is that VEGF in intraocular angiogenesis was markedly upregulated in the aqueous humor and vitreous body [25]. This feature suggests that anti-angiogenic activity by COE was seen in the avascular parts, such as aqueous humor, and vitreous body.

The proliferation and migration of capillary endothelial cells are critical for blood vessel development and are regulated by proangiogenic and antiangiogenic agents [26]. VEGF plays a major role in stimulating endothelial cell proliferation and migration and acts a crucial role in angiogenesis. VEGF expression is known to be inhibited by hyperoxia [27]. Upon termination of hyperoxia exposure, hypoxia-driven vascular proliferation is induced, and VEGF expression is greatly upregulated to a higher level [28, 29]. Several studies suggested that VEGF expression is upregulated in the retina and vitreous of patients or the model animals with ischemic retinopathies [30, 31]. Clinical evidence has suggested that using of VEGF or VEGFR inhibitors, provide great benefits in patients with ROP, neovascular AMD and diabetic retinopathy. The monoclonal anti-VEGF antibody bevacizumab and the tyrosine kinase inhibitors sorafenib and sunitinib are the first FDA approved antiangiogenic drugs that prevent the growth of new blood vessels [7]. However, increasing evidence suggests that AREG, ANG and DLL4 also have a role in retinal neovascularization. Our protein array indicated that COE and BP markedly suppressed the expression of AREG, ANG DLL4 and VEGF. AREG, an epidermal growth factor receptor ligand, is regulated by prolyl-4-hydroxylase domain enzyme 2 (PHD2), a regulator of the stability of HIF-1α that is involved in tumor progression [32] and angiogenesis [33]. AREG has been shown to promote the proliferation of airway epithelial and smooth muscle cells and mucin gene expression [34]. Neutralization of AREG also prevents cultured endothelial cells from forming tubes [35]. ANG has been found to be increased in the vitreous of patients with diabetic retinopathy [36] and contributes to aberrant intrapulmonary neovascularization and remodeling [6]. The DLL4/Notch signaling pathway might play an important role in the HIF-1α-VEGF pathway in the regulation of the progression of CNV under hypoxic conditions [37]. It has also been reported that the up-regulation of DLL4 by VEGF is mediated by both VEGFR1 and VEGFR2, which is dependent on the phosphatidylinositol 3-kinase/Akt pathway but not MAPK/ERK or Src kinases [38].These findings suggest that COE and its bioactive compound, BP elicit antiangiogenic activities by inhibiting the expressions of AREG, ANG and DLL4.

COE is known to have beneficial pharmacological activities in various experimental models. The anti-tumor and anti-metastatic effects of COE might be mediated by its angiogenic activities against neovascularization [39]. COE displays anti-cancer properties by inducing apoptosis via the downregulation of Sp1 in HSC-2 human oral cancer cells and cell cycle arrest in HT29 human colorectal cancer cells [5, 40]. Our results showed that COE and its bioactive compound, BP, exhibited potent efficiency in suppressing HUVEC migration and tube formation. In addition, the effects of COE and BP are mediated not only by VEGF but also by AREG, ANG and DLL4. VEGF plays a major role in retinal neovascularization. However, other angiogenic signaling factors may also be involved in retinal neovascularization. Our previous studies have shown that drugs targeting multiple factors have more potent antiangiogenic activities [15, 41]. Therefore, these observations suggest that COE and BP are promising agents that may block retinal neovascularization by inhibiting multiple angiogenic factors.

Although, BP is a bioactive ingredient of COE, other various compounds, such as ferulic acid, falcarindiol; 6-hydroxy-7-methoxydihydroligustilide; ligustilidiol and senkyunolide H have beed indentified in COE [42]. COE reduced non-perfused areas in the OIR mice, but BP failed to decrease avascular areas. A possible explanation is that herbal extracts are known to have various advantages of synergy and interactions among the various phytochemicals. Yang et al. showed that ferulic acid demonstrated inhibition of endothelial cell proliferation, migration and tube formation [43]. These findings suggest that the inhibitory effect of COE may be a result from synergistic interactions of these compounds.

## Conclusion

COE and BP have potent efficiency in inhibiting endothelial cell migration and tube formation, which are the critical steps in angiogenesis. COE and BP exhibited an antiangiogenic effects by suppressing the expression of AREG, ANG, DLL4 and VEGF in a retinal neovascularization mouse model. Therefore, COE may serve as a valuable herbal medicine in the treatment of human ischemic retinopathy.

## Abbreviations

ANG, angiogenin; AREG, amphiregulin; BP, butylidenephthalide; COE, *Cnidium officinale* Makino; DLL4, delta like ligand 4; HUVECs, human umbilical vein endothelial cells; IGFBP-2, insulin-like growth factor binding protein-2; IL-1α, interleukin-1 alpha; OIR, oxygen-induced retinopathy; TRITC, tetramethylrhodamine isothiocyanate; VEGF, Vascular endothelial growth factor

## References

[1] Campochiaro PA (2013). Ocular neovascularization. J Mol Med (Berl).

[2] Krock BL, Skuli N, Simon MC (2011). Hypoxia-induced angiogenesis: good and evil. Genes Cancer.

[3] Liu Y, Cox SR, Morita T, Kourembanas S (1995). Hypoxia regulates vascular endothelial growth factor gene expression in endothelial cells. Identification of a 5′ enhancer. Circ Res.

[4] Ahn MY, Ryu KS, Lee YW, Kim YS (2000). Cytotoxicity and L-amino acid oxidase activity of crude insect drugs. Arch Pharm Res.

[5] de la Cruz J, Kim DH, Hwang SG (2014). Anti cancer effects of Cnidium officinale Makino extract mediated through apoptosis and cell cycle arrest in the HT-29 human colorectal cancer cell line. Asian Pac J Cancer Prev..

[6] Yao X, Wang W, Li Y, Huang P, Zhang Q, Wang J, Lv Z, An Y, Qin J, Corrigan CJ (2015). IL-25 induces airways angiogenesis and expression of multiple angiogenic factors in a murine asthma model. Respir Res.

[7] Brower V (2009). Antiangiogenesis research is booming, as questions and studies proliferate. J Natl Cancer Inst.

[8] Yeh JC, Cindrova-Davies T, Belleri M, Morbidelli L, Miller N, Cho CW, Chan K, Wang YT, Luo GA, Ziche M (2011). The natural compound n-butylidenephthalide derived from the volatile oil of Radix Angelica sinensis inhibits angiogenesis in vitro and in vivo. Angiogenesis.

[9] Chiu SC, Chen SP, Huang SY, Wang MJ, Lin SZ, Harn HJ, Pang CY (2012). Induction of apoptosis coupled to endoplasmic reticulum stress in human prostate cancer cells by n-butylidenephthalide. PLoS One.

[10] Fu RH, Hran HJ, Chu CL, Huang CM, Liu SP, Wang YC, Lin YH, Shyu WC, Lin SZ (2011). Lipopolysaccharide-stimulated activation of murine DC2.4 cells is attenuated by n-butylidenephthalide through suppression of the NF-kappaB pathway. Biotechnol Lett.

[11] Teng CM, Chen WY, Ko WC, Ouyang CH (1987). Antiplatelet effect of butylidenephthalide. Biochim Biophys Acta.

[12] Ko WC, Liao CC, Shih CH, Lei CB, Chen CM (2002). Relaxant effects of butylidenephthalide in isolated dog blood vessels. Planta Med.

[13] Ko WC, Sheu JR, Tzeng SH, Chen CM (1998). The selective antianginal effect without changing blood pressure of butylidenephthalide in conscious rats. Planta Med.

[14] Mimura Y, Kobayashi S, Naitoh T, Kimura I, Kimura M (1995). The structure-activity relationship between synthetic butylidenephthalide derivatives regarding the competence and progression of inhibition in primary cultures proliferation of mouse aorta smooth muscle cells. Biol Pharm Bull.

[15] Lee YM, Kim CS, Jo K, Sohn EJ, Kim JS, Kim J (2015). Inhibitory effect of Samul-tang on retinal neovascularization in oxygen-induced retinopathy. BMC Complement Altern Med.

[16] Lee YM, Kim J, Jo K, Shin SD, Kim CS, Sohn EJ, Kim SG, Kim JS (2013). Ethyl pyruvate inhibits retinal pathogenic neovascularization by downregulating HMGB1 expression. J Diabetes Res.

[17] Lee YM, Lee YR, Kim JS, Kim YH, Kim J (2015). Cinidium officinale and its Bioactive Compound, Butylidenephthalide, Inhibit Laser-Induced Choroidal Neovascularization in a Rat Model. Molecules.

[18] Henaine-Berra A, Garcia-Aguirre G, Quiroz-Mercado H, Martinez-Castellanos MA (2014). Retinal fluorescein angiographic changes following intravitreal anti-VEGF therapy. J AAPOS.

[19] Stahl A, Connor KM, Sapieha P, Chen J, Dennison RJ, Krah NM, Seaward MR, Willett KL, Aderman CM, Guerin KI (2010). The mouse retina as an angiogenesis model. Invest Ophthalmol Vis Sci.

[20] Stahl A, Connor KM, Sapieha P, Willett KL, Krah NM, Dennison RJ, Chen J, Guerin KI, Smith LE (2009). Computer-aided quantification of retinal neovascularization. Angiogenesis.

[21] Banin E, Dorrell MI, Aguilar E, Ritter MR, Aderman CM, Smith AC, Friedlander J, Friedlander M (2006). T2-TrpRS inhibits preretinal neovascularization and enhances physiological vascular regrowth in OIR as assessed by a new method of quantification. Invest Ophthalmol Vis Sci.

[22] Wang W, Li Z, Sato T, Oshima Y (2012). Tenomodulin inhibits retinal neovascularization in a mouse model of oxygen-induced retinopathy. Int J Mol Sci.

[23] Dorrell MI, Aguilar E, Friedlander M (2002). Retinal vascular development is mediated by endothelial filopodia, a preexisting astrocytic template and specific R-cadherin adhesion. Invest Ophthalmol Vis Sci.

[24] Werdich XQ, McCollum GW, Rajaratnam VS, Penn JS (2004). Variable oxygen and retinal VEGF levels: correlation with incidence and severity of pathology in a rat model of oxygen-induced retinopathy. Exp Eye Res.

[25] Miller JW, Adamis AP, Aiello LP (1997). Vascular endothelial growth factor in ocular neovascularization and proliferative diabetic retinopathy. Diabetes Metab Rev.

[26] Sen CK, Gordillo GM, Khanna S, Roy S (2009). Micromanaging vascular biology: tiny microRNAs play big band. J Vasc Res.

[27] Stone J, Chan-Ling T, Pe’er J, Itin A, Gnessin H, Keshet E (1996). Roles of vascular endothelial growth factor and astrocyte degeneration in the genesis of retinopathy of prematurity. Invest Ophthalmol Vis Sci.

[28] Ashton N (1966). Oxygen and the growth and development of retinal vessels. In vivo and in vitro studies. The XX Francis I. Proctor Lecture. Am J Ophthalmol.

[29] Ashton N (1957). Retinal vascularization in health and disease: Proctor Award Lecture of the Association for Research in Ophthalmology. Am J Ophthalmol.

[30] Aiello LP, Avery RL, Arrigg PG, Keyt BA, Jampel HD, Shah ST, Pasquale LR, Thieme H, Iwamoto MA, Park JE (1994). Vascular endothelial growth factor in ocular fluid of patients with diabetic retinopathy and other retinal disorders. N Engl J Med.

[31] Dorey CK, Aouididi S, Reynaud X, Dvorak HF, Brown LF (1996). Correlation of vascular permeability factor/vascular endothelial growth factor with extraretinal neovascularization in the rat. Arch Ophthalmol.

[32] Chan DA, Kawahara TL, Sutphin PD, Chang HY, Chi JT, Giaccia AJ (2009). Tumor vasculature is regulated by PHD2-mediated angiogenesis and bone marrow-derived cell recruitment. Cancer Cell.

[33] Chan DA, Giaccia AJ (2010). PHD2 in tumour angiogenesis. Br J Cancer.

[34] Berasain C, Avila MA (2014). Amphiregulin. Semin Cell Dev Biol.

[35] Busser B, Sancey L, Brambilla E, Coll JL, Hurbin A (2011). The multiple roles of amphiregulin in human cancer. Biochim Biophys Acta.

[36] Ozaki H, Hayashi H, Oshima K (1996). Angiogenin levels in the vitreous from patients with proliferative diabetic retinopathy. Ophthalmic Res.

[37] Dong X, Wang YS, Dou GR, Hou HY, Shi YY, Zhang R, Ma K, Wu L, Yao LB, Cai Y (2011). Influence of Dll4 via HIF-1alpha-VEGF signaling on the angiogenesis of choroidal neovascularization under hypoxic conditions. PLoS One.

[38] Liu ZJ, Shirakawa T, Li Y, Soma A, Oka M, Dotto GP, Fairman RM, Velazquez OC, Herlyn M (2003). Regulation of Notch1 and Dll4 by vascular endothelial growth factor in arterial endothelial cells: implications for modulating arteriogenesis and angiogenesis. Mol Cell Biol.

[39] Kwak DH, Kim JK, Kim JY, Jeong HY, Keum KS, Han SH, Rho YI, Woo WH, Jung KY, Choi BK (2002). Anti-angiogenic activities of Cnidium officinale Makino and Tabanus bovinus. J Ethnopharmacol.

[40] Lee KE, Shin JA, Hong IS, Cho NP, Cho SD (2013). Effect of methanol extracts of Cnidium officinale Makino and Capsella bursa-pastoris on the apoptosis of HSC-2 human oral cancer cells. Exp Ther Med.

[41] Lee YM, Kim CS, Sohn E, Jo K, Lim HR, Kim SK, Kim JS, Kim J (2014). Sipjeondaebo-tang, a traditional herbal formula, inhibits retinal neovascularization in a mouse model of oxygen-induced retinopathy. Tohoku J Exp Med.

[42] Bae KE, Choi YW, Kim ST, Kim YK (2011). Components of rhizome extract of Cnidium officinale Makino and their in vitro biological effects. Molecules.

[43] Yang GW, Jiang JS, Lu WQ (2015). Ferulic acid exerts anti-angiogenic and anti-tumor activity by targeting fibroblast growth factor receptor 1-mediated angiogenesis. Int J Mol Sci.

